# Effect of Ionizing Radiation on the Transcript Variants Expression of *p21* Gene

**DOI:** 10.31557/APJCP.2021.22.11.3717

**Published:** 2021-11

**Authors:** Amir Danyaei, Ali Teimoori, Hashem Khanbabaei, Halime Mansoury Asl

**Affiliations:** 1 *Department of Medical Physics, School of Medicine, Ahvaz Jundishapur University of Medical Sciences, Ahvaz, Iran. *; 2 *Department of Virology, Faculty of Medicine, Hamedan University of Medical Sciences, Hamedan, Iran. *; 3 *Department of Radiologic Technology, Faculty of Allied Medicine, Kerman University of Medical Sciences, Kerman, Iran.*; 4 *Student Research Committee, Ahvaz Jundishapur University of Medical Sciences, Ahvaz, Iran. *

**Keywords:** p21, irradiation, transcript variants, cyclin-dependent kinase inhibitor1

## Abstract

**Purpose:**

CDK1A is one of the most important genes that have different key roles in cell lines. This gene has several transcript variants. Investigating of expression of each one actually can be so important because any one of them may have a separate unknown role in cancer cells so can be used to increase therapeutic efficacy.

**Methods:**

A549, MDA–MB–231 and Hek–AD cell lines were used in this study. Firstly, three primers for variants of p21 gene were designed by Snapgene and BLAST software. Secondly, the variants expression was checked for each cell lines by RT–qPCR technique, separately. Then the variants that expressed in the cells were selected for more investigation. Finally 2 Gy irradiation was used to evaluate the effect of that on variants expression.

**Results:**

The results show that for all cell lines, primer num1 and 3 expressed before any stimuli. After irradiation, for MDA–MB–231 and A549, the expression of primer num3 was decreased, while for Hek–AD no change was observed. The primer num1 expression after the irradiation was different for the cells, V1 expression was decreased in A549 by fold of 0.03 while expression of this for MDA–MB–231 cells was not changed after 2Gy irradiation.

**Conclusion:**

It is very necessary to pay attention to the function of each splice variant as well as the response to external stimuli. Understanding the role of each variant in a gene is critical and researchers can use that to improve radiotherapy as well.

## Introduction

The generation of transcript variants by alternative splicing is a common process in human gene expression; indeed, >70% of human genes are known to express transcript variants (Johnson et al., 2003). Various studies have shown that the transcript variants of a gene may have different and even opposing roles, and alternative splicing is known to be a key factor in cancer progression. For example, Bcl-x, which is associated with cell survival/apoptosis, has two isoforms, Bcl-xL and Bcl-xS. The longer Bcl-xL isoform acts as an apoptotic inhibitor, whereas the shorter form acts as an apoptotic activator (Shkreta et al., 2008). In prostate adenocarcinoma, transcripts V4 and V1 were significantly upregulated but only transcript V4 upregulation was associated with poor recurrence-free survival in The Cancer Genome Atlas data (Xiao et al., 2020). Splice variant (sv) AC333 inhibited MCF7 cell proliferation. Similarly, svAC333 knockdown by RNA interference was shown to have the opposite effect by repressing the cell cycle progression of breast cancer cells (Yuan et al., 2020). 

One of the most important genes in cells is *p21*. This gene encodes a potent cyclin-dependent kinase inhibitor. The CDK inhibitor p21, also known as p21waf1/cip1 or cyclin-dependent kinase inhibitor1A (Abbas and Dutta, 2009a), belongs to the Cip/Kip family of cdk inhibitors (Harper et al., 1993). *CDKN1A* gene located on chromosome 6 (6p21.2) in humans (Gartel, 2006). 

Using immunohistochemistry, recent studies has revealed that p21 was expressed in a variety of human malignancies, and is correlated with tumor progression and a poor prognosis in various carcinomas (Chen et al., 2013; Xu et al. 2013). Evidence exists that p21 is an important factor implicated in clinicopathological features and predicated the prognosis of several cancer such as bladder (Liukkonen et al., 2000), breast (Zohny et al., 2017; Sanaei and Kavoosi, 2020), colorectal (Song et al., 2015), prostate (Sivoňová et al., 2015) cancer. There are several treatment modalities including radiotherapy with ionizing radiation (IR) for cancer. In response to IR, p53 protein is activated and it has various downstream targets including genes involved in cell-cycle regulation, apoptosis, and DNA repair. Regulation of these processes by p53 controls the cellular response to IR-induced damage. As soon as DNA is damaged by radiation, binding of p53 protein induces transcription of the downstream gene *p21*, which stops cells from entering into the S-phase (El-Deiry et al., 1994).

p21 is encoded by ten transcript variants in humans (Radhakrishnan et al., 2006). The difference of transcript variants is in their 5’ UTR s but are similar in their coding. Vogel et al suggested that translation regulation contributes to the protein variation as several parameters related to translation like 5′ UTR, 3′ UTR, coding sequence length, presence of uORFs and amino acid composition, and so forth showed good correlations with the obtained mRNA/protein ratios (Vogel et al., 2010).

Studies have been done on the role of various p21 variants (Lehman et al. 2015; Joseph et al., 1998; Collier et al., 2018). However, according to our search in several databases, there is little strong information about the regulation of CDKN1A expression via its transcript variants. While regulation of p21 expression is known, the regulation of the individual transcript variants is Unknown. Furthermore, the effects of transcript variants expression has not been extensively studied. External stimuli such as ionizing radiation have a significant effect on gene expression and will cause noteworthy changes in cell proliferation cycle.

Radiotherapy is a cancer treatment that uses high doses of radiation to kill cancer cells and shrink tumors. When used to treat cancer, radiation therapy can cure cancer, prevent it from returning, or stop or slow its growth. But in some cancer cells caused radioresistance. By knockdown or knockout a transcript variant of the responsible gene, we can improve radiotherapy efficiency. In the First step, to access this goal, it is necessary to examine how the expression of each variant changes in response to ionizing radiation and what effect it has on the cells.

The purpose of this study was to explore transcript variants expression of *p21* gene after ionizing radiation. We believe understanding of the function and behavior of transcript variants of *p21* gene, with two controversy roles, is so important and effectiveness. These results can help researchers and oncologists for target and irradiation therapy and finally improve therapeutic efficiency. We investigated the changes in the expression of p21 variants in three different cell lines followed by 2Gy irradiation..

## Materials and Methods

Cell lines. MDA–MB–231breast cancer, A549 lung cancer and Hek–AD Human embryonic kidney cell lines were purchased from the Iranian Biological Resource Center (Tehran, Iran) and was cultured in Dulbecco’s Modified Eagle’s Medium (Gibco; Thermo Fisher Scientific, Inc., Grand Island, NY, USA) supplemented with 10% FBS (Gibco; GE Healthcare Life Sciences, Chalfont, UK) and 5% penicillin/ streptomycin. Cultures were maintained at 37°C with 5% CO2 in a humidified incubator.

Reverse transcriptionquantitative polymerase chain reaction (RTqPCR): At first, the* p21* gene sequence were collected from the https://www.ncbi.nlm.nih.gov. Then BLAST and Snapgene softwares were used for designing each primer individually, and after checking primer design by Oligo and gene Runner programs, ordered for synthesis. Total RNA was extracted using RNeasy Plus-Mini Kit (Qiagen, Hilden, Germany) according to the manufacturer’s protocol. The same amount of RNA quantified via NanoDrop One (Thermo Fisher Scientific, Waltham, MA) was used to synthesize complementary DNA using QuantiTect Reverse Transcription Kit (Qiagen). Finally, the QuantiNova SYBR Green PCR Kit (Qiagen) was used to run the real-time quantitative polymerase chain reaction (RT-qPCR) using LightCycler 96 System (Roche, Basel, Switzerland).The mRNA expression level was quantified by normalizing over HPRT as the internal control gene. The sequences of the primers used for RT-qPCR were is shown in table 1. 

Fold of difference relative to the reference gene (HPRT) was determined by conversion of 2^−∆∆CT^. ∆∆CT = (CT objective gene – CT reference gene) of experimental group − (CT objective gene – CT reference gene) of control group.

Irradiation strategy. For more investigation and evaluated the regulation of expressed variants, the cells were exposed to 2Gy X-ray using a linear accelerator (LINAC) which produces X photons of 6 Mev at a dose rate of 200 mGy/min (Varian, Golestan hospital, Ahwaz, Iran). The SSD was 100cm, and the field size was 10*10 cm.

Statistical analysis. All experiments were repeated for at least three times. Data and statistics are presented as means ± SD. The significance was determined by One Way ANOVA using SPSS 24.0. P<0.05 was considered to indicate a statistically significant difference (*P<0.05; **P<0.01).

## Results

Briefly, in this study, three different cell lines were chosen. MDA–MB–231 breast cancer cell line, A549 lung cancer cell line and Hek–AD human embryonic kidney cell line. Firstly, according to last Refseq update in www.ncbi.nlm.nih.gov, three specific primers were designed. Primer number 1 (num1) transcribes V1 (NM_000389.5) and V5 (NM_001220777.2), primer num2 transcribes V4 (NM_001220778.2), and primer num3 transcribes V2 (NM_078467.3), V3 (NM_001291549.3), and V6 (NM_001374509.1) of the* p21* gene. Secondly, after validation of primers synthesis, expression of these primers was investigated in the cell lines, separately. Finally, we used 2Gy x-ray ionizing irradiation, a routine dose that is used in radiotherapy, as an external stimulus to explore the effect of ionizing irradiation on the expression of p21 transcript variants. Transcript variant 4 of the* p21* gene is not expressed.

As mentioned in the Materials and Methods section, RT–qPCR technique was used to examine the expression of the p21 variants. It was found that for all cell lines, primers num 1 and 3 were expressed, but num2 was not expressed in MDA–MB–231, A549, and Hek–AD cell lines. So, V1, 5 (primer num1), V2, 3, 5, and 6 (primer num 3) are expressed in the cell lines. But V4 (primer num2) is not expressed in these.

Irradiation has different effects on the expression of p21 variants. The cells were exposed to 2Gy irradiation. After 24h, expression of primers num 1 and 3 was examined.

**Figure 1:**
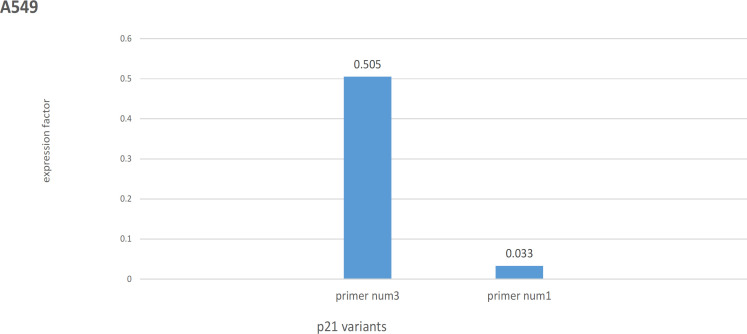
Irradiation Decreased the Variants Expression in A549 Cell Lline (P<0.01). Primer number 1 (num1) transcribes V 1 and 5, primer num3 transcribes V2, 3, and 6 of the p21 gene

RT–qPCR analysis for A549 showed that irradiation significantly decreased the expression of the primer num 1, 3 by a fold of 0.03 and 0.50, respectively (Figure 1). In other words, the expression of V1, 2, 3, 5, and 6 of the *p21* gene has decreased in this cell line after irradiation.

**Figure 2:**
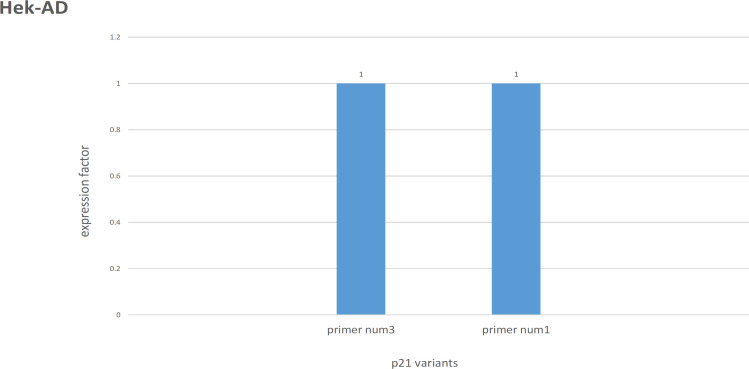
Irradiation NOT Changed Variants Expression in Hek–AD Cell Line (P>0.05)

Interestingly, for Hek–AD, primers expression was not changed 24h after 2Gy irradiation (p>0.05) (Figure 2). 

**Figure 3:**
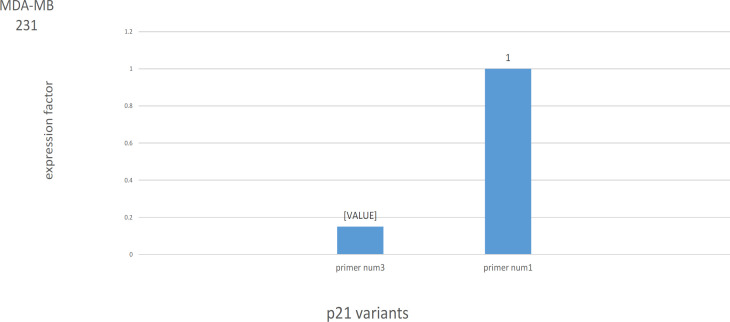
Irradiation Decreased Primer num3 Expression in MDA–MB–231 Cell Line (P<0.01)

The results of RT–qPCR for another cancer cell line, MDA–MB–231 breast cancer cell line, are shown in Figure 3. Notably, we observed that the expression of primer num3 was down-regulated (0.15 fold). But expression of primer num1 was not changed after irradiation (p>0.05). It means that irradiation has not to effect on V1 but caused downregulation in V 2, 3, 5, and 6 transcript variants of the* p21* gene.

**Table 1:** Primers Sequences

Gene name	Forward seq (5’-3’)	Tm	Reverse seq (5’-3’)	Tm	product length
p21.num1	GCCGAAGTCAGTTCCTTGTG	59.13	TTCTGACATGGCGCCTCCT	61.29	84
p21- num2	GTTCACAGGTGTTTCTGCGGC	62.58	CCGCCATTAGCGCATCACA	61.18	146
p21- num3	AACATGTTGAGCTCTGGCATAGAA	60.57	GTTCTGACATGGCGCCTGAAAA	61.96	80
HPRT	TAG CCC TCT GTG TGC TCA AG	59.39	ACT TTT ATG TCC CCT GTT GAC TG	58.6	160

## Discussion

p21 is known as an inhibitor of cell cycle. This gene includes different variants. Investigating of expression of each one actually can be so important in cancer researches because any one of them may have a separate unknown role in cancer cells. In in vitro researches, primers are designed often expressed all variants altogether.

In all of studies checked, the primers was applied to all p21 variants. Furthermore, not enough attention has been paid to the different roles of each variant in a gene. Consequently, the effect of external stimuli especially irradiation, which is used in radiotherapy, on transcript variants expression on the cancer cells has not been strongly investigated.

The difference of transcript variants is in their 5’ UTRs and variations in 5’ UTR can function as important switches to regulate gene expression (Araujo et al., 2012). 

For example, variant (1) of the *p21* gene differs in the 5’ UTR, lacks an in-frame portion of the 5’ coding region, and initiates translation at a downstream start codon, compared to variant 3 of this gene. 

In this study, three different cell lines were chosen. A549 lung cancer cell line, MDA–MB–231 breast cancer cell line, and Hek–AD human embryonic kidney cell line.MDA–MB–231 and A549 are two aggressive cell lines in breast and lung cancer and often radioresistance was observed in these cells. MDA–MB–231 cell line is ER, PR, and E-cadherin negative, triple-negative, invasive, and expresses mutated p53. Also, radiation resistance is often seen in this.Hek–AD human embryonic kidney cell line that is used as a normal cell line. The expression of variants of the* p21* gene was investigated. In three different cell lines (MDA–MB–231, A549, and Hek–AD), three different primers, for variants of *p21* gene, were designed. In the first, expression of each one was separately examined. 

The results showed that for all cell lines, variant primers num 1, 3 were expressed. 

Indeed we observed that V1, 2, 3, 5, and 6 are expressed in the cell lines. But V4 (primer num2) is not expressed in these. This is a notable result and the question appears why variant 4 is not expressed in these three cell lines although this variant, like the variants 1, 2, and 5, which expressed in these cells, encodes isoform 1 in the CDK1A.In the other word, Is variant 4 not expressed in certain cells, including the cells was used in our study, or is it silenced in all human cells (cancer and normal)?

P21 is one of the most important gene in cells that has certain and key roles including: regulator of cell cycle progression at G1, cell cycle G1 phase arrest in response to a variety of stress stimuli, and also apoptosis following caspase activation (Kho et al., 2004). p21 is a two-faced regulator depending on cell type, cellular localization, p53 status, and the type and level of genotoxic stress. It can acquire either oncosuppressive or oncopromoting properties depending on whether it is in a p53-proficient or p53-deficient environment, respectively (Georgakilaset al., 2017). Nozell and Chen (2002) found that a single locus, p21, can express two unique gene products, both of which appear to have a role in cell cycle regulation. 

In second step of this study, the cells received 2Gy irradiation to evaluate the effect of ionizing irradiation on variants expression. 24h after that, the expression of V1, 2, 3, 5, and 6 (expressed primer in step one) was investigated. Interestingly, the expression of these variants was different in the cells after irradiation. In A549 cell line, a decrease in primer num1 expression was observed. While, for MDA–MB–231 there was no difference in this before and after irradiation. It means that V1 of the *p21 *gene downregulated in lung cancer cell line but in an aggressive breast cancer cell line no change was observed. (To confident that this results are not accidentally, we tripled RT–qPCR and observed the same outcomes). Reports of Collier et al show that just V1 expression was detected in N-TERT cells.We can attribute this observed difference to two different reasons: First, the dissimilarity in the different cancer cell lines has caused this variance. On the other hand, as mentioned above, the *p21* gene has different roles in cells (repair, apoptosis, G1 arrest). After studying Hek–AD, the results of RT–qPCR showed that no variant expression has changed after 2 Gy irradiation. Therefore, the response to radiotherapy in different cells can be partially justified. X-ray radiotherapy causes cell resistance or cell sensitivity in different cells. Actually, the expression of genes, followed by the expression of proteins in cells, creates these features. However, different functions of the *p21* gene can be relate to the different expression of variants in cells. The proteins expression will be necessary to evaluate in others research according to these evidences and can be an interested issue in future studies. High expression of p21 protein in head/neck, esophageal, and oral squamous cell carcinomas has been correlated with poor prognosis, indicating that loss of CDKN1A regulation could promote cancer progression in epithelial cells (Rigberg et al., 1998). Although CDKN1A expression is often dysregulated in a variety of cancers, its expression has been shown to be either increased or decreased depending on the cancer and cell type (Abbas and Dutta, 2009b). Therefore, therapies targeting CDKN1A have proven problematic (Eastman et al., 2004), and targeting key signaling factors regulating CDKN1A expression may help circumvent these complexities. 

In our study we observed that stressors such as IR (in this research) and UVB (Collier et al., 2018) have different effects on the variants expression and as highlighted above, it is certainly because of difference in the cell lines. All of this conclusions provide necessity of the attention to p21 splice variants as an important gene for cancer therapy.

These features can be used in target therapy to increase the therapeutic efficiency of radiotherapy. By knocking down or knocking out only one or more variants of the *p21* gene, that are responsible for causing cell repair in this, we can increase the radiation sensitivity of cancer cells and did not change the normal cells or even increase their radiation resistance.

In this study, Prime num3 expression (V2, 3, 5, and 6) decreased in two cancer cell lines. MDA–MB–231 and A549 are very aggressive and radiation-resistant among their cell lines. We next wished to investigation of the cell cycle distribution in the cells to evaluate that there is any relation between expressions of these variants with regulation of cells cycle (data not published).

In conclusion, the importance of gene and target therapy for cancer treatment is clear for all and likewise, it is proven that each cell line has a specific response to stressors that is due to several genes function. Primer designing is one of the most important in biology. It is very necessary to pay attention to the function of each splice variant as well as the response to external stimuli. Understanding the role of each variant in a gene is critical and researchers should be careful when designing it. Because, as the results of this study show, irradiation has caused different responses in different p21 variants in the cell lines. We have chosen the *p21* gene as the most important gene in cells spatially in cancer cells. Our results showed when primers were designed it is eventually necessary to design on different variants of a gene, separately. So researchers can accurately consider the responses of cells and obtain the best treatment for each cancer with target therapy. However, the p21 variants require more exploration in different cells and proteins expression.

## Author Contribution Statement

Halime MANSOURY ASL: collected the data, wrote the paper. Amir DANYAEI: wrote the paper, performed the analysis. Ali TEIMOORI: conceived and designed the analysis. Hashem KHANBABAEI: collected the data

## Availability of data and material

All data of this study is documented and will be provided to the journal if necessary.

## Conflict of interest

There is no conflict of interest.
